# Impact of boron and indium doping on the structural, electronic and optical properties of SnO_2_

**DOI:** 10.1038/s41598-021-92450-2

**Published:** 2021-06-22

**Authors:** Petros-Panagis Filippatos, Nikolaos Kelaidis, Maria Vasilopoulou, Dimitris Davazoglou, Alexander Chroneos

**Affiliations:** 1 Institute of Nanoscience and Nanotechnology (INN), National Center for Scientific Research Demokritos, 15310 Agia Paraskevi, Athens, Greece; 2 Faculty of Engineering, Environment and Computing, Coventry University, Priory Street, Coventry, CV1 5FB UK; 3 Department of Materials, Imperial College, London, SW7 2AZ UK

**Keywords:** Condensed-matter physics, Materials for devices, Theory and computation

## Abstract

Tin dioxide (SnO_2_), due to its non-toxicity, high stability and electron transport capability represents one of the most utilized metal oxides for many optoelectronic devices such as photocatalytic devices, photovoltaics (PVs) and light-emitting diodes (LEDs). Nevertheless, its wide bandgap reduces its charge carrier mobility and its photocatalytic activity. Doping with various elements is an efficient and low-cost way to decrease SnO_2_ band gap and maximize the potential for photocatalytic applications. Here, we apply density functional theory (DFT) calculations to examine the effect of p-type doping of SnO_2_ with boron (B) and indium (In) on its electronic and optical properties. DFT calculations predict the creation of available energy states near the conduction band, when the dopant (B or In) is in interstitial position. In the case of substitutional doping, a significant decrease of the band gap is calculated. We also investigate the effect of doping on the surface sites of SnO_2_. We find that B incorporation in the (110) does not alter the gap while In causes a considerable decrease. The present work highlights the significance of B and In doping in SnO_2_ both for solar cells and photocatalytic applications.

## Introduction

Tetragonal SnO_2_ is a wide bandgap semiconductor, which typically exhibits n-type conductivity due to the oxygen vacancies that are created during the crystallization process^1–4^. SnO_2_ is commonly used for glazes^1^, polishing powder^2^, photovoltaics^3^, and gas sensors^4^. Recently, SnO_2_ has been demonstrated as a cathode material in Li-ion batteries^5^. Wang et al.^5^ have shown that Sn_2_O_3_ in SnO_2_ nanosheets significantly improves battery performance. However, the common SnO_2_ films exhibit low intrinsic carrier mobility, which has been attributed to deep donor states originating from oxygen vacancies^3^. Many doping strategies have been adopted to further increase its electrical conductivity and improve its absorption in the visible region—for photocatalytic applications—by reducing its wide energy gap. Specifically, many experimental and theoretical works have demonstrated that halogen doping in SnO_2_ increases conductivity and transparency, making it a better candidate for optoelectronic applications^6,7^.

Another option for improving the properties the properties of SnO_2_ is doping with p-type elements^8^. Transparent conducting oxides (TCOs) fabricated from doped semiconductor oxides such us In:SnO_2_ (ITO), F:SnO_2_ (FTO) and B:ZnO (BZO) are commonly used as transparent conductive materials in industrial applications such as displays and lighting devices^8^. In these structures, the metal atom is typically substituted by the dopant, which improves the charge carrier conductivity. Experimental reports on mesoporous oxides indicate remarkable chemical properties when B occupies an interstitial position^9^. These B interstitials (B_i_) enhance the carrier densities of the semiconductors and lead to a diverse coordination environment with hyperstoichiometric oxygen vacancies. Additionally, some reports propose B as a doping candidate for SnO_2_ based photocatalytic applications^10^. In particular, B atoms can occupy interstitial positions creating a hybridization of the B-p orbital with the nearest O-2p orbital^11^. This improves the activity of catalysts as it reduces the e^−^/h^+^ recombination^12^. Tran et al.^13^ studied the optical and the electrical properties of B: SnO_2_ revealing that the transmittance of the films is increased with the increase of the B dopant concentration. This translates into a decrease of the band gap as compared to undoped SnO_2_, which make the material applicable for photocatalytic devices. Interestingly, they reported the dependance of the transmittance with the crystallization temperature, which can be attributed to the increased scattering of photons due to the larger grains as well as the increase of tin and oxygen vacancies. Regarding the electrical properties of the material, it was determined that B doping beneficially increases the carrier concentration compared to the undoped SnO_2_^13^.

Indium is an important dopant used to increase the electrical and optical properties of SnO_2_^14–16^. Aouaj et al.^14^ compared the electrical and optical properties of ITO and FTO and they determined that at high In content the absorbance is lower in ITO and the gap value is increased. Additionally, they predicted that that the electrical resistivity is generally lower in ITO as compared to FTO. Finally, they concluded that ITO at different In concentration can be used as a transport layer or electrode in solar cells. Similar experiments of Kulkarni et al.^15^ showed that the deposition temperature and substrate play a major role regarding the optical properties of ITO and variation to the refractive index value.

Apart from the experimental works, there are also theoretical studies based on DFT that examine the influence of doping in SnO_2,_ mostly in substitutional positions^16–24^. Especially for the halogen substitutional doping, it is revealed that single donor states arise inside the bandgap; however, the effect of interstitials is not generally taken into consideration by the community. Mallick et al.^23^ examined theoretically the impact of substitutional Al in SnO_2_. They showed that the introduction of aluminium (Al) leads to new defect states above the Fermi energy level which are highly depend on the doping concentration. The band gap of Al:SnO_2_ remains unchanged for low doping concentrations (below 1.85%) while there is a small increase in high concentrations (higher than 3.70%). Duan et al.^24^ investigated the effect of gallium (Ga) in SnO_2_ and determined that Ga substitutionals in Sn sites, shifts the Fermi energy to the valence band and introduces more charge holes at the Ga sites. The Ga doping also slightly reduces the band gap and increases the electrical conductivity of the material. Nevertheless, the effect of B and In incorporation in SnO_2_ is not extensively examined using computational modelling techniques. From all the above it is concluded that the B family group elements (group IIIA of the periodic table) introduce energy states in the band gap which may play a major role in photovoltaic and photocatalytic devices.

In most previous DFT studies, the bandgap of SnO_2_ is considerably underestimated and predicted at a value of 1–2 eV^22,25^. This is a well-known deficiency of the Local Density Approximation (LDA) and of the Generalized Gradient Approximation (GGA). Therefore, the use of computationally demanding but more accurate hybrid exchange–correlation functionals are deemed necessary to accurately describe the band gap and the position of states in the gap or at its edges.

In the present study, we apply Hybrid functional DFT calculations using PBE0^45^ to attain a bandgap value of 3.35 eV, which agrees well with the experimentally reported bandgap^3,26^. More analytically, we examine the effect of B and In doping on the bulk SnO_2_ and we also investigate the effect of interstitial doping of the (110) surface for the first time. Density of states (DOS) calculations showed a reduction of the band gap in all the substitutional cases and the formation of energy states in the bandgap for the substitutional and interstitial doping. This bandgap reduction combined with the created inter-gap energy levels are highly beneficial for the photocatalytic applications of SnO_2_ while the predicted characteristics can also be applied in photovoltaic technologies.

## Results

### Bulk rutile SnO_2_

**Figure 1:**
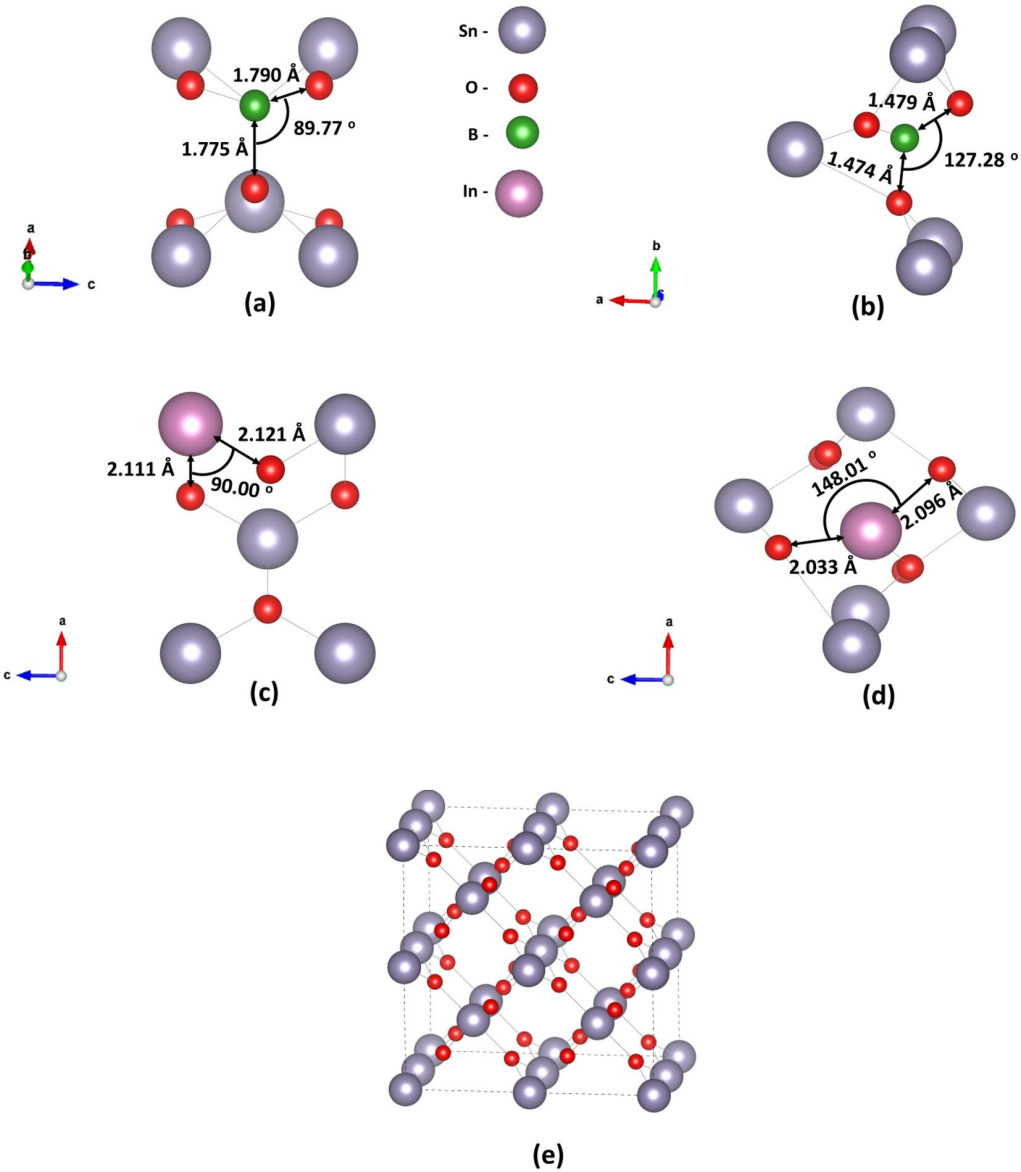
The structures of (**a**) boron substitutional doped SnO_2_ (B_Sn_:SnO_2_), (**b**) boron interstitial doped SnO_2_ (B_i_:SnO_2_), (**c**) indium substitutional doped SnO_2_ (In_Sn_:SnO_2_), (**d**) indium interstitial doped SnO_2_ (In_i_:SnO_2_) and (**e**) the bulk supercell.

SnO_2_ forms in the P4_2_/mmm space group crystalizing in the rutile structure. The experimental unit cell parameters a = b = 4.737 Å and c = 3.186 Å were determined using X-ray diffraction (XRD) experiments^16^. The calculated lattice parameters after the relaxation of rutile SnO_2_ are a = b = 4.717 Å and c = 3.189 Å in very good agreement with the experimental values. The dopant percentage of the present calculations is 1 B or In atom in 48 SnO_2_ atoms, which results in 2.08% doping. Zhang et al*.*^27^ performed experiments regarding the effect of B in tin oxide and they predicted that B can either be at a Sn substitutional site or occupy an interstitial site. Zhang et al*.*^27^ determined that with this amount of doping the thickness of the film is reduced in parallel with the mean crystal size. We predict that in its Sn substitutional site the B atom is at a distance of 1.775 Å from the nearest oxygen atom (refer to Fig. 1a) while for the interstitial case, the boron atom is at a distance of 1.475 Å from the O-atom (refer to Fig. 1b). In Fig. 1c,d we show the In_Sn_:SnO_2_ and In_i_:SnO_2_ cases, respectively. The substitutional dopant is relaxed at a distance of 2.11 Å from the nearest oxygen atom, while the interstitial is located at a distance of 2.03 Å. In Fig. 1e we present the bulk supercell used. In Table 1, we report the relevant lattice parameters and volume changes, through doping, for the bulk system. Concerning the B:SnO_2_ cases, there is a small decrease in the lattice volume for the substitutional case, which can be attributed to the smaller ionic radius of B than Sn (B^3+^: 0.23 Å, Sn^4+^: 0.71 Å) while for the interstitial case, there is an increase. The present results agree with the available experimental data that predict a decrease of the lattice parameters for the B_Sn_:SnO_2_ and an increase for the B_i_:SnO_2_^9^. Similarly for In:SnO_2_ we predicted that in all the cases, there is an increase to the lattice parameters. This is in agreement with previous experimental studies^28^.

**Table 1:** The calculated lattice constants and cell volumes for all the dopants.

	a (Å)	c (Å)	Vol (Å^3^)
SnO_2_	4.717	3.189	70.956
B_Sn_:SnO_2_	4.674	3.153	68.881
B_i_:SnO_2_	4.866	3.225	76.361
In_Sn_:SnO_2_	4.730	3.194	71.459
In_i_:SnO_2_	4.901	3.220	77.343

**Figure 2:**
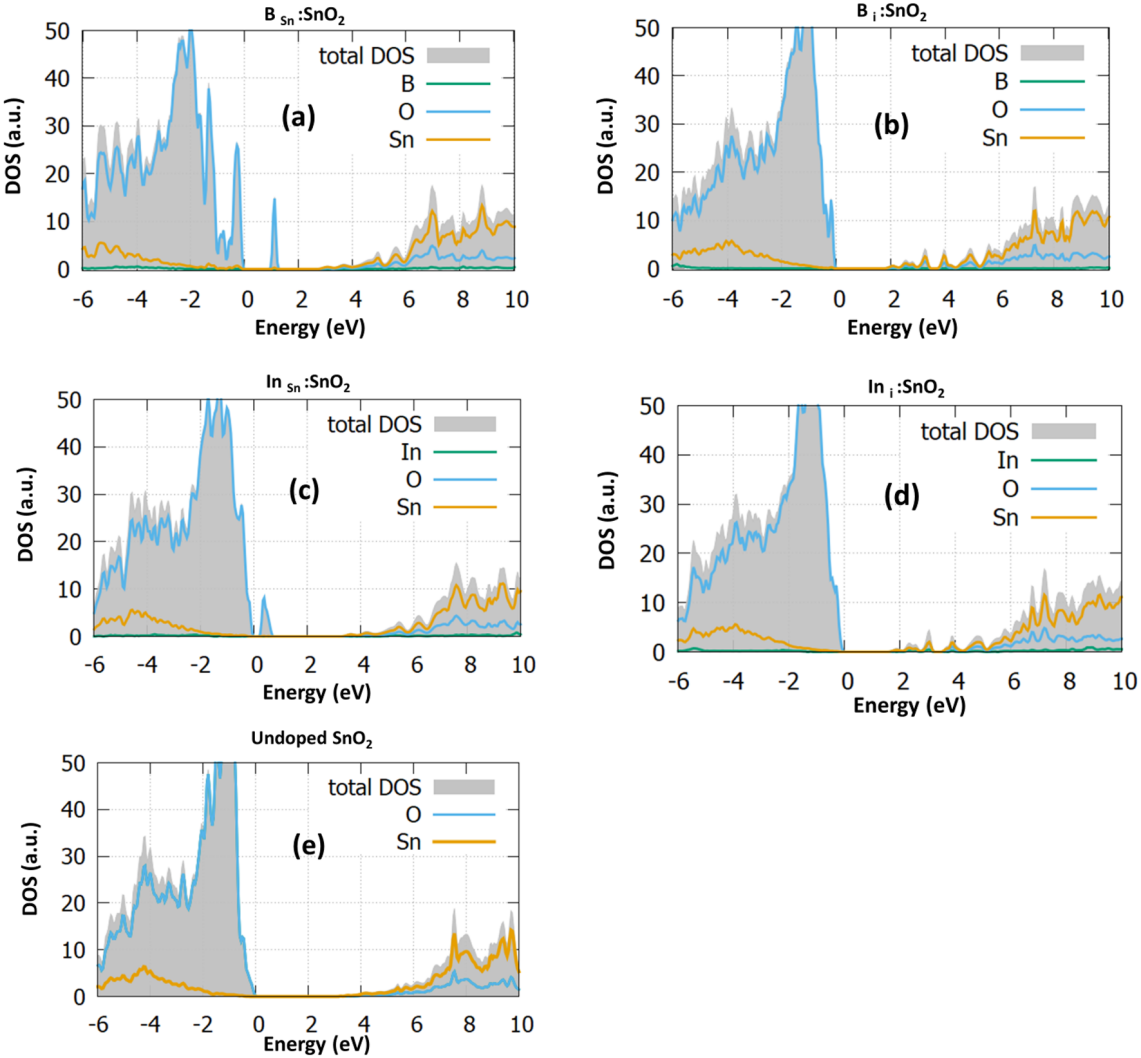
The total density of states (DOS) and the projected density of states (PDOS) of (**a**) B_Sn_:SnO_2_, (**b**) B_i_:SnO_2_, (**c**) In_Sn_:SnO_2_, (**d**) In_i_:SnO_2_ and (**e**) undoped SnO_2_.

For each doping case, we calculate the DOS and the PDOS and present them in Fig. 2. The DOS of the undoped SnO_2_ is given in Fig. 2e for reference. To have a clear and more reliable picture of the changes of the electronic structure due to doping, we employed the hybrid functional PBE0, which provides a good bandgap value (calculated at 3.35 eV) and is in agreement with the experimental value^26^.

In Fig. 2a, the B_Sn_:SnO_2_ case is examined. In this case, it is observed from the total DOS (grey) that the bandgap is reduced to the value of 2.73 eV while some energy states are created inside the bandgap at 1 eV. Furthermore, it is seen that some additional states arise at the valence band maximum. Looking at the PDOS of the B substitutional case, we predict that these states are created mostly from the hybridization of O-2p with Sn-5p and the B-2p orbitals. This is in agreement with the work of Yu et al*.*^29^, which also predicts mid-gap states and gap reduction when B is inserted. However, in their study, there is an underestimation to the bandgap value of SnO_2_. Experimental studies^30^ have shown that 2% B doping decreases the bandgap, which is in good agreement with the present study.

To further investigate the effect of B doping, we considered interstitial B in SnO_2_. We expect that the interstitial incorporation of B results in gap states forming to the conduction band edge between 1.66 and 3.49 eV. The bandgap is seen to increase to the value of 3.72 eV. As it is discussed in previous studies, the formation of energy states in the middle of the bandgap is highly beneficial for photocatalytic applications, but still, they can be a crucial disadvantage for PV and LED devices as they work as “traps” which reduce the device photocurrent and the photogenerated charge carriers^30,31^. Although B_i_:SnO_2_ has not been examined theoretically, Zhi et al*.*^9^ predicted that interstitial atomic positions play a significant role in increasing the capacity of tin oxide, making it a good fit for supercapacitor applications. Zhi et al*.*^9^ used 4% B and they concluded that the interstitial atoms slightly increased the bandgap. This is the Moss-Burstein effect and it is generally seen in heavily doped semiconductors, where after a doping concentration, the bandgap of the semiconductor starts to increase and can even reach higher values than the undoped material^32^. In the case of B_i_:SnO_2,_ this provides a sufficient number of charge carriers that improve the capacitance of SnO_2_^33^. We predicted a bandgap increase in the case of the bulk SnO_2_ while in the (110) surface of SnO_2_ the gap remained unchanged. The (110) plane is considered the most highly energetic plane of the experimental SnO_2_ structure^9,34,35^ as we discuss in the next section. In essence the present calculations complete the DFT work of Zhi et al*.*^9^, which focused only to the (002) plane. Zhang et al.^27^ also performed electrical measurements to predict the properties of B:SnO_2_. They predicted that in the interstitial position, B releases three free electrons resulting in an increase of the free electron concentration. Tran et al.^13^ predicted that the bandgap of SnO_2_ is decreased with the B incorporation and with the increase of the temperature. While this is opposite to what we predicted or other experimental works^9^ we believe that this is due to the effect of intrinsic defects, such as oxygen vacancies, which affect the gap value. Also, the percentage of B atoms that reside in substitutional sites play an important role to the increase of the transmission of light within SnO_2_. However, it is clear that even in this case the Moss-Burstein effect is still seen as the band gap is increasing after the 4 at.% doping percentage.

Continuing with the bulk In:SnO_2_ we examined the interstitial and substitutional formation. Focusing on the DOS of In_Sn_:SnO_2_ (Fig. 2c) we predicted again unoccupied energy states near the valence band at 0.8 eV, which are created due to the hybridization of O-2*p* with Sn-5*p* and In-5*p*. These gap states might be beneficial for device fabrication as they will serve as a route for the transition from the valence to the conduction band. Furthermore, now the bandgap is slightly decreased at a value of 3.25 eV. Thereafter, we performed calculations for the interstitial doping of indium in SnO_2_. We predict that the In-5*p* orbitals play a significant role in shifting the conduction band and reducing the band gap, which reaches a value of 3.48 eV. Similarly to the B case, the interstitial doping gives rise to energy levels inside the gap in the area between 1.48 and 3.29 eV. The present results agree well with the experimental data of Abdulsattar et al*.*^36^, which predicted a slight decrease to the bandgap with a doping percentage of 2%. All these results show that although B:SnO_2_ shows energy states that are detrimental for device applications, it is evident that gap reduction makes it possible for photocatalytic applications. In contrast, In:SnO_2_ shows characteristics that make it a good candidate for an electron transport layer material for photovoltaic devices.

**Figure 3:**
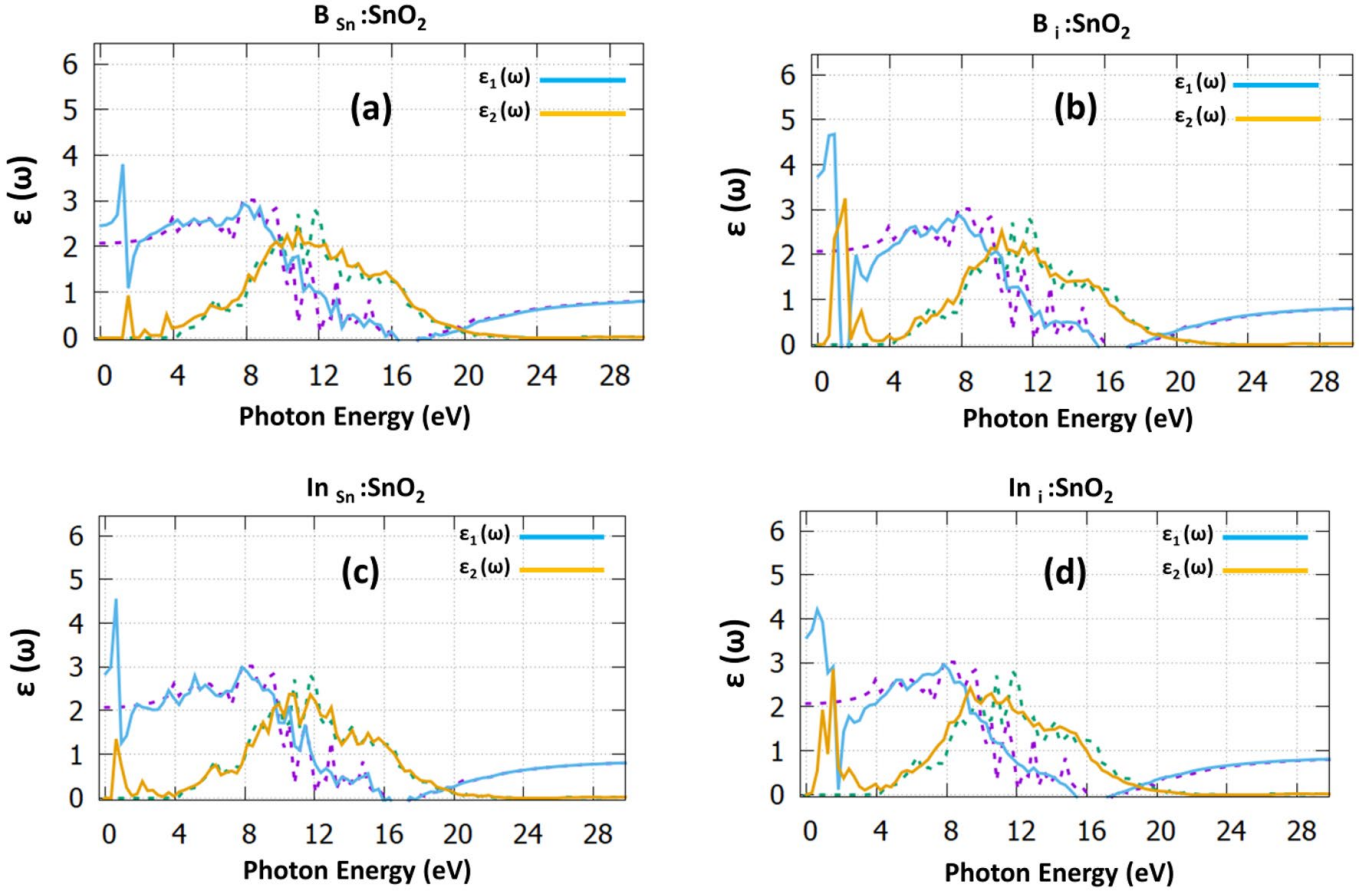
The dielectric function for (**a**) B_Sn_:SnO_2_, (**b**) B_i_:SnO_2_, (**c**) In_Sn_:SnO_2_ and (**d**) In_i_:SnO_2_. The dotted purple and dotted green, correspond to the dielectric function of the undoped SnO_2_.

To consider possible applications of the doped bulk structures, we calculated their optical properties. In order to describe the absorption and dispersion mechanisms from the occupied to the empty electron levels, we predicted the dielectric function, presented in Fig. 3. Focusing on the real part of the dielectric function for the undoped SnO_2_ (dotted purple), it is seen that it reaches a maximum value of 8.5 eV. The negative value at 16.5 eV is attributed to the metallic properties of undoped SnO_2_^37,38^. The imaginary part of the dielectric function provides helpful information regarding the probability of photon absorption. We calculated that there is a decreasing trend for all the cases due to Maxwell–Wagner interfacial polarization^39^. From the undoped SnO_2_ case (dotted green), two sharp peaks arise due to the transitions from O-2*p* to Sn-5*p*. The dielectric constant of the undoped case is predicted at 2 in good agreement with previous experimental^37^ and theoretical works^38^. Focusing on B_Sn_:SnO_2_ (refer to Fig. 3a) case, we predicted the dielectric constant is significantly increased to the value of 2.5. The major peak that arises at 1.7 eV of the imaginary part can be attributed to the transition of electrons of B-2*p* and Sn-5*p*. Continuing with the interstitial B case (refer to Fig. 3b), there is a considerable increase to the dielectric constant, which reaches a value of 3.8.

Regarding the In_Sn_:SnO_2_ (Fig. 3c) the dielectric constant is increased to the value of 3, which is significantly higher than the B_Sn_:SnO_2_ and finally, for the In_i_:SnO_2_ (Fig. 3d) the dielectric constant reaches the value of 3.7. From Fig. 3 it can also be observed that there is a shift of the low photon energy peaks towards the visible region accompanied by an increase of the value of the dielectric constant. The recombination of charge carriers decreases, where the static dielectric constant monitors the electric fields inside the material by polarization. As a result, both B_i_:SnO_2_ and In_i_:SnO_2_ are highly preferred to use as heterojunction buffer layers in photovoltaic applications.

**Figure 4:**
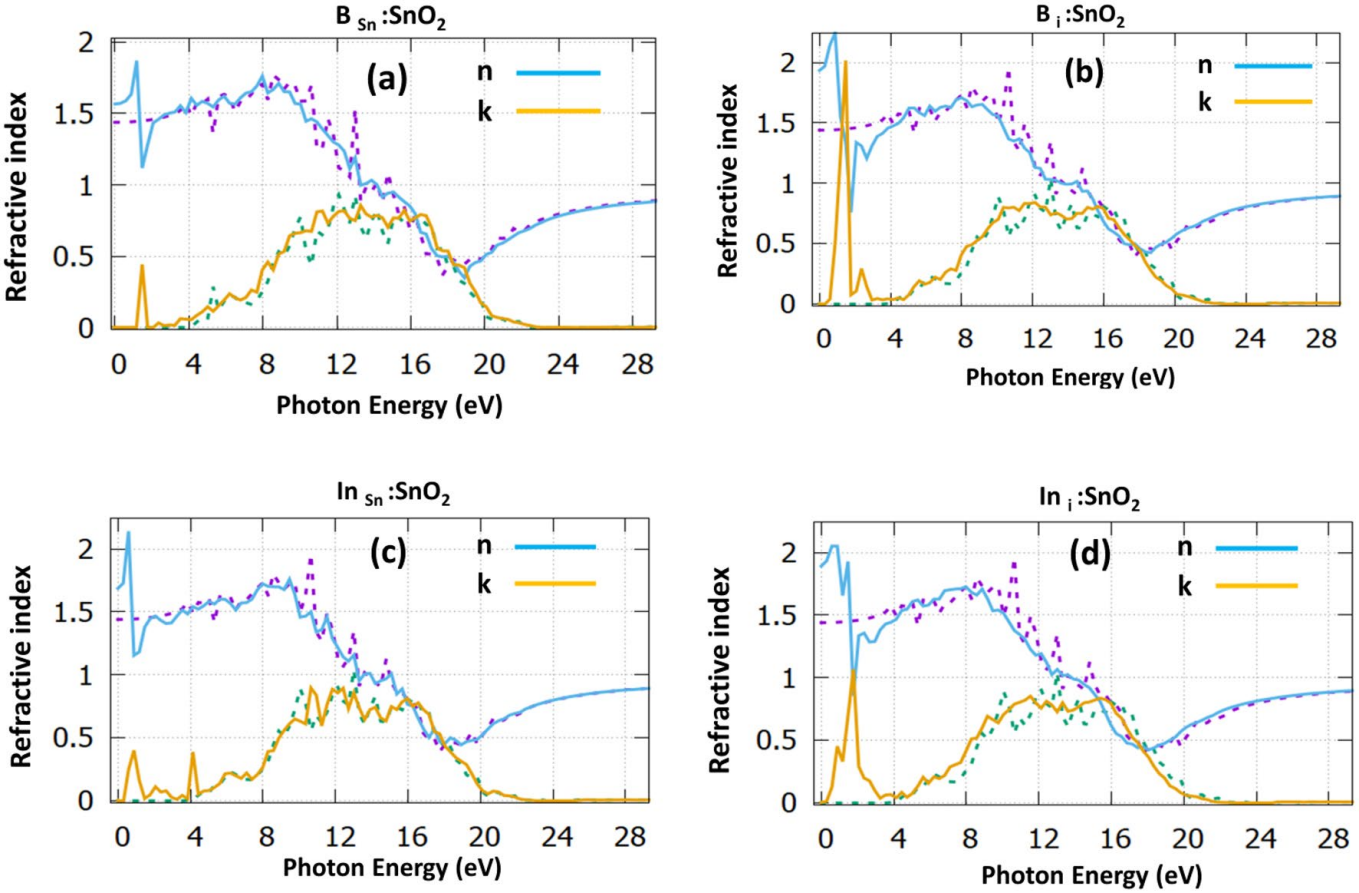
The refractive index for (**a**) B_Sn_:SnO_2_, (**b**) B_i_:SnO_2_, (**c**) In_Sn_:SnO_2_ and (**d**) In_i_:SnO_2_. The dotted purple and dotted green, correspond to the dielectric function of the undoped SnO_2_.

The refractive index is shown in Fig. 4. For the SnO_2_ the refractive index is computed in zero frequency at the value of 1.40, which agrees with previous simulation studies^38^, but it is underestimated compared to the experimental value of 1.70^40^.

Regarding the doping structures, we predicted a value of 1.55 for B_Sn_:SnO_2_ (Fig. 4a), while for B_i_:SnO_2_ (Fig. 4b), there is an increase reaching a value of 1.95. Considering In doping, we predicted a value of 1.6 for the substitutional case (Fig. 4c) and a value of 1.85 for the interstitial In (Fig. 4d). It is evident that for the refractive index, there is an increase in the lower photon energies and on the contrary, there is a significant decrease in the upper energies. This is due to the optical dispersion that these structures have.

**Figure 5:**
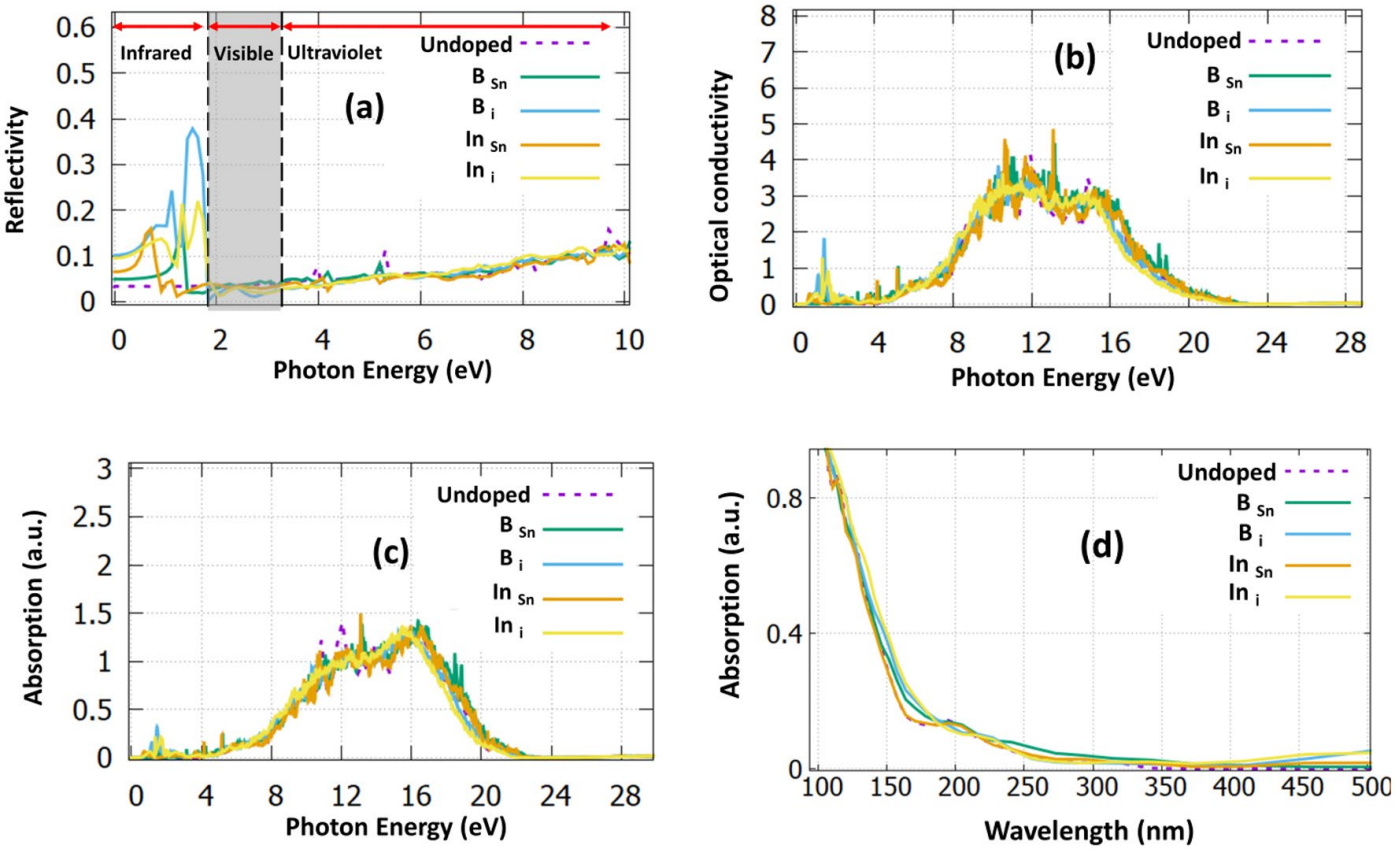
(**a**) The reflectivity for all the doped structures (**b**) The optical conductivity for all the studied structures (**c**) the absorption coefficient for all the structures versus the photon energy (**d**) the absorption coefficient for all the structures versus the wavelength. The dotted purple and dotted green, correspond to the undoped SnO_2_.

To provide a further insight into the optical properties of all these structures, in Fig. 5 we calculated the reflectivity, the optical conductivity, and the absorption of the structures. Reflectivity (see Fig. 5a) is an important property of these materials as it signifies the amount of photons that are reflected from the material. We have shown that the values at zero photon energy are calculated at 3%, 5%, 10%, 6% and 9.5% for SnO_2_, B_Sn_:SnO_2_, B_i_:SnO_2_, In_Sn_:SnO_2_ and In_i_:SnO_2_ respectively. We predicted that B_i_:SnO_2_ has the highest reflectivity in the near-infrared and visible regions compared to all the other cases. Importantly, all the examined cases encounter less than 15% reflectivity in the infrared and visible region; thus they can be used as antireflective coatings when the dopant concentration is at 2%^41^. In Table 2 we have gathered all the calculated electrical and optical constants for reference.

**Table 2:** The calculated electronic and optical constants.

	Bandgap (eV)	Dielectric constant	Refractive index	Reflectivity
SnO_2_	3.33	2.0	1.40	0.03
B_Sn_:SnO_2_	2.73	2.5	1.55	0.05
B_i_:SnO_2_	3.72	3.8	1.95	0.1
In_Sn_:SnO_2_	3.25	3	1.60	0.06
In_i_:SnO_2_	3.48	3.7	1.85	0.095

In Fig. 5b we present the optical conductivity of all the calculated structures. If a photon has higher energy than the optical bandgap then a transition occurs and an electron–hole pair (exciton) is generated. The mobility of these excitons represent the optical conductivity, which is an important parameter that is used to design optical detectors^42^. Due to the electronic charge neutrality, these excitons do not contribute to the electrical conductivity^43^. The highest excitonic features are calculated at 13.2 eV for undoped, B_Sn_:SnO_2_ and In_Sn_:SnO_2_ cases while for the interstitial dopants they are shifted to 9 eV and 10 eV for the B and In, respectively. The optical conductivity also describes the losses for a wave with the same frequency. From the graph, we can conclude that SnO_2_ and In_i_:SnO_2_ have lower losses than the other cases. Lastly, in Fig. 5c,d we present the absorption co-efficiency. The absorption for the undoped case starts at 380 nm, which is underestimated compared to the experimental value, which is approximately 400 nm^44^. It is seen that In_i_ and B_i_ have the highest absorption to the visible region.

From the above, we can conclude that B_i_ and In_i_ doped SnO_2_ exhibits interesting characteristics, constituting the material applicable to photovoltaic and photocatalytic devices.

### Surface (110) of rutile SnO_2_

As Zhi et al*.*^9^ indicated to further understand the effect of B_i_, the surface of SnO_2_ needs to be examined. For applicable photocatalytic materials with a visible light response and high charge mobility, such as SnO_2_, attention to the high-energy surfaces should be paid when the structure is doped with atoms or molecules. The studies of SnO_2_ surface are significantly less than the studies of the bulk and they primarily focus on sensor applications^45^. In this section, the change to the electronic properties of B and In doped SnO_2_ will be investigated. Here we used a slab model with a vacuum of 12 Å thickness across the (110) plane. We chose this particular surface because it is the most intense crystallization plane^9,45^ of rutile SnO_2_ and it has never been studied before for B or In doping. Furthermore, as the (110) plane is one of the most highly energetic surfaces, it will play the role of the active site in photocatalytic reactions^46^. Indium doping improves the electrical characteristics in SnO_2_ nanowire structures. Specifically, the field enhancement factor is higher compared to other dopants^47–49^. In these applications, the surface of the nanowire plays a significant role and it is responsible for the fluctuations in the field emission properties^47^.

Here we predicted the interstitial position of both B and In by examining many different configurations and keeping the lowest energy system. To accurate predict the electronic characteristics, we used the hybrid functional PBE0.

**Figure 6:**
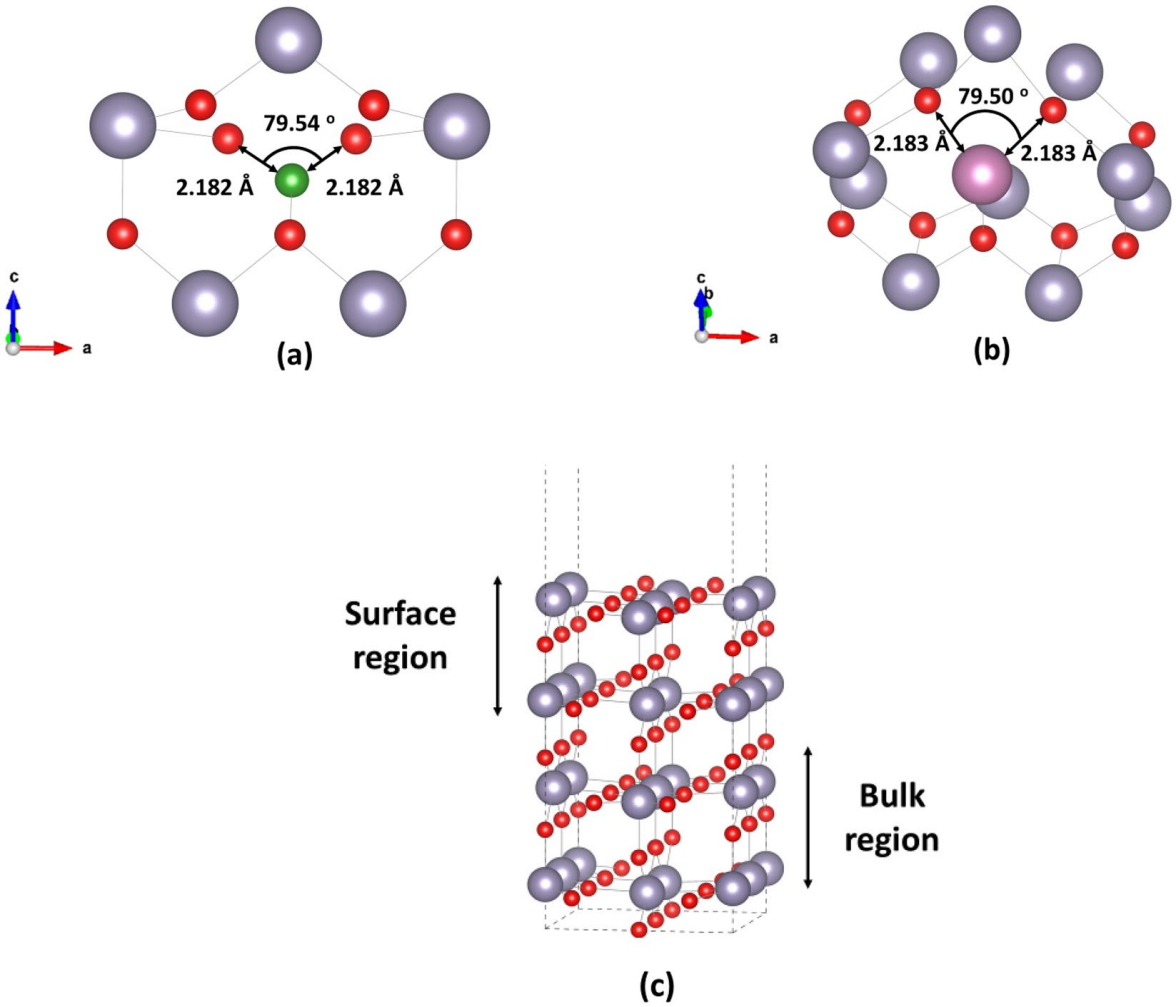
The structures of (**a**) boron doped (110) SnO_2_ surface, (**b**) indium doped (110) SnO_2_ surface and (**c**) the (110) surface supercell.

**Figure 7:**
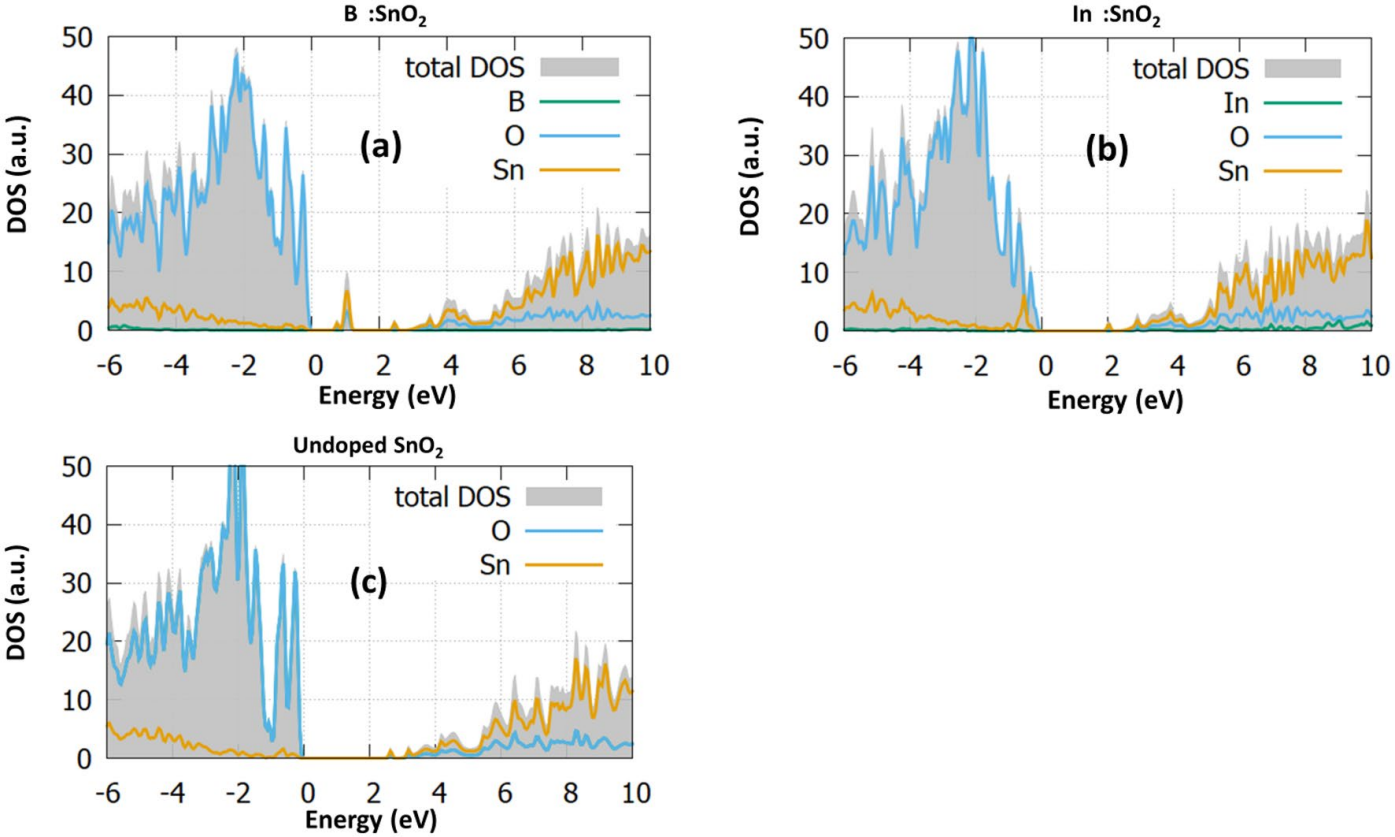
The total density of states (DOS) and the projected density of states (PDOS) of (**a**) B (**b**) In and (**c**) undoped (110) SnO_2_ surface.

In Fig. 6a,b we present the B_i_:SnO_2_ and In _i_:SnO_2_ doping cases while in Fig. 6c the supercell for the (110) plane is presented for reference. The boron interstitial displaces the tin atom and occupies a tin site to minimize its energy (Fig. 6a). We predicted that interstitial boron sites at 2.182 Å from the nearest oxygen atom and 3.10 Å from the nearest tin atom. On the other hand, we calculated that In atom sites at a distance of 2.183 Å from the nearest oxygen atom and 3.32 Å from the nearest tin atom. Looking at the DOS and the pDOS of Fig. 7a, it is seen that boron interstitial produces a small band peak at 1 eV.

Interestingly in this high-energy plane the bandgap is not decreased compared to the undoped case (see Fig. 7c). We believe that this result combined with the bulk B_i_:SnO_2_ explains the experimental phenomenon that Zhi et al.^9^ discussed in their paper about B_i_. Looking at the In_i_:SnO_2_ (refer to Fig. 7b) we can see that the (110) surface bandgap decreases to 2 eV. Indium doping of the surface SnO_2_ improves the sensitivity of tin oxide based gas sensors as it significantly affects the crystallization of the samples^50^. The gap of the undoped (110) SnO_2_ is shown in Fig. 7c at 2.5 eV, a good agreement with previous DFT studies^51^. All the above results indicate that In doping in the SnO_2_ shows good characteristics that should be examined in photocatalytic applications.

## Conclusions

In the present study, we applied DFT calculations to investigate the electronic and optical properties of boron and indium doped bulk and surface SnO_2_. In particular, we performed DOS calculations and we found that the bulk structures have a bandgap increase for interstitial boron and indium doping. Conversely, for the substitutional cases, the bandgap is notable decreased. Mid-gap states are formed in all the examined cases. Although these states might be detrimental for photovoltaics as they may act as trap states for photogenerated carriers, they can be useful for photocatalytic applications based on B:SnO_2_ and In:SnO_2_. The present study explains some of the phenomena that have been experimentally observed and paves the way for applications of boron and indium structures to more applications such as supercapacitors or sensors. Regarding the indium defect, we predicted that at low concentrations highly wanted states are formed near the valence band, which can be beneficial for energy harvesting devices. Furthermore, its optical characteristics and the bandgap reduction at the surface make it a worthy candidate for photocatalysis.

## Methodology

We employed the Cambridge Serial Total Energy Package (CASTEP)^52,53^. For our calculations we used the hybrid functional PBE0 in order to consider the effect of the localized electrons and the bandgap underestimation that is generally encountered in GGA and LDA^54^. The cutoff energy was chosen at 800 eV after performing the convergence test and for our calculations we used a 48 atom supercell (2 × 2 × 2 unit cells) with 2 × 2 × 3 k-points for the sampling of the Brillouin zone during the geometry optimization^54^. To predict the interstitial positions in all cases, we used geometry optimization and we examined all possible configurations. Finally, we kept the position, which provides the lowest energy system. For the simulation of the surface we used a slab model with a vacuum of approximately 12 Å vertical to the (110) direction. In our system, the top 2 layers represent the surface while the bottom 2 are fixed and mimic the bulk region. For the DOS calculations we used a k-point mesh of 5 × 5 × 5 for the bulk modelling while for the surface we applied a 3 × 3 × 1 set. Finally, the convergence criteria for our simulations were chosen at 2.0 ∙ 10^−5^ eV/atom for the SCF tolerance, 0.05 eV/Å for the force tolerance and 0.001 Å for the Max displacement tolerance.

## References

[1] Searle AB (1935). The Glazer’s Book.

[2] Holleman AF, Wiberg E (2001). Inorganic Chemistry.

[3] Greenwood NN, Earnshaw A (2012). Chemistry of the Elements.

[4] Tountas M, Topal Y, Kus M, Ersöz M, Fakis M, Argitis P, Vasilopoulou M (2016). Water-soluble lacunary polyoxometalates with excellent electron mobilities and hole blocking capabilities for high efficiency fluorescent and phosphorescent organic light emitting diodes. Adv. Funct. Mater..

[5] Wang C, Du G, Ståhl K, Huang H, Zhong Y, Jiang JZ (2012). Ultrathin SnO_2_ nanosheets: Oriented attachment mechanism, nonstoichiometric defects, and enhanced lithium-ion battery performances. J. Phys. Chem. C.

[6] Rakhshani AE, Makdisi Y, Ramazaniyan HA (1998). Electronic and optical properties of fluorine-doped tin oxide films. J. Appl. Phys..

[7] Filippatos PP, Kelaidis N, Vasilopoulou M, Davazoglou D, Chroneos A (2021). Defect processes in halogen doped SnO_2_. Appl. Sci..

[8] Minami T (2005). Transparent conducting oxide semiconductors for transparent electrodes. Semicond. Sci. Technol..

[9] Zhi J, Zhou M, Zhang Z, Reiser O, Huang F (2021). Interstitial boron-doped mesoporous semiconductor oxides for ultratransparent energy storage. Nat. Commun..

[10] Kong XB, Li F, Qi ZN, Qi L, Yao MM (2017). Boron-doped tin dioxide films for environmental applications. Surf. Rev. Lett..

[11] Zhao W, Ma WH, Chen CC, Zhao JC, Shuai ZG (2004). Efficient degradation of toxic organic pollutants with Ni2O3/TiO_2__−__x_B_x_ under visible irradiation. J. Am. Chem. Soc..

[12] Bagwasi S, Tian B, Zhang J, Nasir M (2013). Synthesis, characterization and application of bismuth and boron co-doped TiO_2_: A visible light active photocatalyst. Chem. Eng. J..

[13] Tran QP, Fang JS, Chin TS (2016). Optical properties and boron doping-induced conduction-type change in SnO_2_ thin films. J. Electron. Mater.

[14] Aouaj MA, Diaz R, Belayachi A, Rueda F, Lefdil MA (2009). Comparative study of ITO and FTO thin films grown by spray pyrolysis. Mater. Res. Bull..

[15] Kulkarni AK, Lim T, Khan M, Schulz KH (1998). Electrical, optical, and structural properties of indium-tin-oxide thin films deposited on polyethylene terephthalate substrates by RF sputtering. J. Vac. Sci. Technol. A.

[16] Peng-Fei L, Yue S, Zhong-Yuan Y, Long Z, Qiong-Yao L, Shi-Jia LM, Li-Hong H, Yu-Min L (2012). Electronic structure and optical properties of antimony-doped SnO_2_ from first-principle study. Commun. Theor. Phys..

[17] Canestraro CD, Roman LS, Persson C (2009). Polarization dependence of the optical response in SnO_2_ and the effects from heavily F doping. Thin Solid Films.

[18] Rivera R, Marcillo F, Chamba W, Puchaicela P, Stashans A (2013). SnO_2_ physical and chemical properties due to the impurity doping. Lect. Notes Eng. Comp..

[19] Velikokhatnyi OI, Kumta PN (2011). Ab-initio study of fluorine-doped tin dioxide: A prospective catalyst support for water electrolysis. Phys. B.

[20] Golovanov V, Golovanova V, Kuisma M, Rantala TT (2013). Electron spin resonance parameters of cation vacancies in tin dioxide doped with fluorine and hydrogen. J. Appl. Phys..

[21] Oshima M, Yoshino K (2012). Structural and electronic structure of SnO_2_ by the first-principle study. Trans Tech Stäfa.

[22] Govaerts K, Partoens B, Lamoen D (2016). Extended homologous series of Sn–O layered systems: A first-principles study. Solid State Commun..

[23] Mallick HK, Zhang Y, Pradhan J, Sahoo MPK, Pattanaik AK (2021). Influence of particle size and defects on the optical, magnetic and electronic properties of Al doped SnO_2_ nanoparticles. J. Alloys Compd..

[24] Duan Y, Zheng J, Fu N, Hu J, Liu T, Fang Y, Zhang Q, Zhou X, Lin Y, Pan F (2015). Effects of Ga doping and hollow structure on the band-structures and photovoltaic properties of SnO_2_ photoanode dye-sensitized solar cells. RSC Adv..

[25] Perdew JP, Levy M (1983). Physical content of the exact Kohn–Sham orbital energies: Band gaps and derivative discontinuities. Phys. Rev. Lett..

[26] Zervos M, Lathiotakis N, Kelaidis N, Othonos A, Tanasa E, Vasile E (2019). Epitaxial highly ordered Sb:SnO_2_ nanowires grown by the vapor liquid solid mechanism on m-, r- and a-Al_2_O_3_. Nanoscale Adv..

[27] Zhang B, Tian Y, Zhang JX, Cai W (2011). The structural and electrical studies on the Boron-doped SnO_2_ films deposited by spray pyrolysis. Vacuum.

[28] Lekshmy SS, Joy K (2014). Structural and optoelectronic properties of indium doped SnO_2_ thin films deposited by sol gel technique. J. Mater. Sci. Mater. Electron..

[29] Yu J, Wang Y, Huang Y, Wang X, Guo J, Yang J, Zhao H (2020). Structural and electronic properties of SnO_2_ doped with non-metal elements. J Nanotechnol..

[30] Filippatos PP, Kelaidis N, Vasilopoulou M, Davazoglou D, Lathiotakis NN, Chroneos A (2019). Defect processes in F and Cl doped anatase TiO_2_. Sci. Rep..

[31] Filippatos PP, Soultati A, Kelaidis N, Petaroudis C, Alivisatou AA, Drivas C, Kennou S, Agapaki E, Charalampidis G, Yusoff ARM, Lathiotakis NN, Coutsolelos AG, Davazoglou D, Vasilopoulou M, Chroneos A (2021). Preparation of hydrogen, fluorine and chlorine doped and co-doped titanium dioxide photocatalysts: A theoretical and experimental approach. Sci. Rep..

[32] Butcher K, Hirshy H, Perks RM, Wintrebert M, Chen PPT (2006). Stoichiometry effects and the Moss-Burstein effect for InN. Phys. Status Solidi A.

[33] Simon P, Gogotsi Y (2008). Materials for electrochemical capacitors. Nat. Mater..

[34] Kumar M, Kumar A, Abhyankar AC (2014). SnO_2_ based sensors with improved sensitivity and response-recovery time. Ceram. Int.

[35] Manassidis I, Goniakowski J, Kantorovich LN, Gillan MJ (1995). The structure of the stoichiometric and reduced SnO_2_ (110) surface. Surf. Sci..

[36] Abdulsattar MA, Batros SS, Addie AJ (2016). Indium doped SnO_2_ nanostructures preparation and properties supported by DFT study. Superlattices Microstruct..

[37] Tingting S, Fuchun Z, Weihu Z (2015). Density functional theory study on the electronic structure and optical properties of SnO_2_. Rare Metal Mater. Eng..

[38] Khan AF, Mehmood M, Aslam M, Ashraf M (2010). Characteristics of electron beam evaporated nanocrystalline SnO_2_ thin films annealed in air. Appl. Surf. Sci..

[39] Singh A, Chatterjee R, Mishra SK, Krishna PSR, Chaplot SL (2012). Origin of large dielectric constant in La modified BiFeO_3_–PbTiO_3_ multiferroic. J. Appl. Phys..

[40] Afify HH, Momtaz RS, Badawy WA, Nasser SA (1991). Some physical properties of fluorine-doped SnO_2_ films prepared by spray pyrolysis. J. Mater. Sci. Mater. Electron..

[41] Khoshman JM, Kordesch ME (2006). Optical properties of a-HfO_2_ thin films. Surf. Coat. Technol..

[42] Akinlami JO, Olateju IO (2012). Reflection coefficient and optical conductivity of gallium nitride GaN. Quant. Electron. Optoelectron..

[43] Dash LK, Vast N, Baranek P, Reining MC, Cheynet L (2004). Electronic structure and electron energy-loss spectroscopy of ZrO_2_ zirconia. Phys. Rev. B.

[44] Doyan A, Susilawati, Imawanti YD (2017). Synthesis and characterization of SnO_2_ THIN layer with a doping aluminum is deposited on quartz substrates. AIP AIP Conf. Proc..

[45] Wang X, Qin H, Chen Y, Hu J (2014). Sensing mechanism of SnO_2_ (110) surface to CO: Density functional theory calculations. J. Chem. Phys. C.

[46] Talebian N, Jafarinezhad F (2013). Morphology-controlled synthesis of SnO_2_ nanostructures using hydrothermal method and their photocatalytic applications. Ceram. Int..

[47] Bhise AB, Late DJ, Walke P, More MA, Mulla IS, Pillai VK, Joag DS (2007). A single In-doped SnO_2_ submicrometre sized wire as a field emitter. J. Phys. D: Appl. Phys..

[48] Bhise AB, Late DJ, Walke PS, More MA, Pillai VK, Mulla IS, Joag DS (2007). Sb-doped SnO_2_ wire: Highly stable field emitter. J. Cryst. Growth.

[49] Bhise AB, Late DJ, Ramgir NS, More MA, Mulla IS, Pillai VK, Joag DS (2007). Field emission investigations of RuO_2_-doped SnO_2_ wires. Appl. Surf. Sci..

[50] Cui S, Wen Z, Mattson EC, Mao S, Chang J, Weinert M, Hirschmugl CJ, Gajdardziska-Josifovska M, Chen J (2013). Indium-doped SnO_2_ nanoparticle–graphene nanohybrids: Simple one-pot synthesis and their selective detection of NO_2_. J. Mater. Chem. A.

[51] Li M, Zhu H, Wei G, He A, Liu Y (2019). DFT calculation and analysis of the gas sensing mechanism of methoxy propanol on Ag decorated SnO2 (110) surface. RSC Adv..

[52] Segall MD, Lindan PJ, Probert MA, Pickard CJ, Hasnip PJ, Clark SJ, Payne MC (2002). First-principles simulation: Ideas, illustrations and the CASTEP code. J. Phys. Cond. Matter.

[53] Ceperley DM, Alder BJ (1980). Exchange-correlation potential and energy for density-functional calculation. Phys. Rev. Lett..

[54] Paier J, Marsman M, Hummer K, Kresse G, Gerber IC, Angyan JG (2006). Screened hybrid density functionals applied to solids. J. Chem. Phys..

